# Effects of Hybridization and Triploidization on Transcription of Core Metabolic and Stress Response Genes in Rainbow Trout (*Oncorhynchus mykiss*) × Brook Trout (*Salvelinus fontinalis*) Hybrids—Preliminary Results

**DOI:** 10.3390/cimb48030320

**Published:** 2026-03-17

**Authors:** Marcin Kuciński, Rafał Rożyński, Konrad Ocalewicz

**Affiliations:** 1 Department of Marine Biology and Biotechnology, Faculty of Oceanography and Geography, University of Gdansk, M. Piłsudskiego 46 Av., 81-378 Gdynia, Poland; konrad.ocalewicz@ug.edu.pl; 2 Department of Salmonid Research, National Inland Fisheries Research Institute in Olsztyn, Rutki, 83-330 Żukowo, Poland; r.rozynski@infish.com.pl

**Keywords:** aquaculture, hybridization, triploidization, gene expression, salmonid fishes

## Abstract

The transcriptomic effects of hybridization and triploidization were investigated in diploid and triploid rainbow trout, diploid brook trout, as well as triploid hybrids of rainbow trout and brook trout. The examined fish were reared under identical conditions for about two and a half years after hatching. Expression of ten genes involved in cellular respiration (*Atp5bp*, *Slc25a5*), mitochondrial functioning (*Mrpl28*, *Micu2*), ribosome biogenesis (*Rpl24*, *Rps24*), proteasome-mediated protein turnover (*Derl1*, *Psmc2*), and protein chaperoning (*Hsp90B1*, *Pdia4*) was studied in liver and muscle tissues. Most of the analyzed genes (*Atp5bp*, *Slc25a5*, *Mrpl28*, *Micu2*, *Rpl24*, *Rps24*, *Derl1,* and *Psmc2*) displayed comparable expression levels in the liver tissue across the examined triploid hybrids and diploid parental species, with stabilization of genes that were both positively and negatively compensated in the triploid rainbow trout. In turn, significant upregulation of *Slc25a5*, *Derl1*, *Rps24,* and *Rpl24* genes, together with downregulation of *Micu2* gene, was observed in the triploid rainbow trout liver and muscle, respectively. On the other hand, triploid hybrids showed marked transcriptional upregulation of genes primarily associated with energy metabolism and protein synthesis (*Atp5pb*, *Slc25a5*, *Rpl24*, *Rps24,* and *Pdia4*) relative to all the fish groups examined. Although protein-synthesis- and energy-related genes were upregulated in the muscles of triploid hybrids, the recorded growth performance data did not indicate clear evidence of growth heterosis (MPH = −14.3% for body weight; MPH = −0.4% for body length), suggesting that potential benefits of increased heterozygosity in this cross may not be fully reflected in enhanced growth. Three- to four-fold downregulation of the heat shock protein (*Hsp90B1*) gene was also observed in both tissues of triploid hybrids compared with purebred diploid and triploid trout, which may reflect potential maladaptive genomic effects commonly observed in distant salmonid crosses, suggesting altered stress-response regulation in the examined triploid hybrids.

## 1. Introduction

Distant hybridization is a genetic breeding technique widely used in aquaculture to produce fish with enhanced or new traits that are more suitable for commercial production and better aligned with customer preferences [1,2,3]. Integration of genomes from different species results in rapid changes to both genotype and phenotype, often triggering hybrid heterosis [4,5,6]. As a result, distant fish crosses may exhibit higher growth rates, enhanced resistance to pathogens, increased tolerance to unfavorable environmental conditions, improved meat quality, and more attractive appearance [7,8,9,10,11]. There is, nevertheless, a negative relationship between hybrid fitness and the accumulation of neutral or adaptive genetic differences between the parental genomes, making the crossing of different species not only an opportunity for trait superiority but also a challenge, as it usually leads to the hybrid breakdown [12,13,14].

Another reproductive biotechnical technique utilized in aquaculture is triploidization [15]. Triploid development is artificially induced by exposing fertilized eggs to chemical or physical shocks (i.e., sub-lethal temperature or high hydrostatic pressure), which prevents the extrusion of the second polar body from the egg [16]. The nuclear genome of the resulting embryos comprises three (3n) sets of chromosomes (two from the female and one from the male) [17]. The odd number of chromosomes in triploid fish disrupts gonadal development and gamete production, often resulting in functional sterility. This, in turn, eliminates the negative effects of sexual maturation on growth rate, meat quality, and overall fitness compared to diploid individuals [18,19]. Production of sterile triploids also mitigates potential ecological risks posed by escaped cultured fish from farms or by their introduction into natural watersheds for recreational purposes [20,21]. On top of that, numerous studies have highlighted that triploidization can improve the viability of distant fish hybrids [5,11,15,22].

Apart from their aquaculture potential, triploid hybrids should be considered as models in studies of gene expression regulation. Recent studies on cyprinids indicate that, in triploid hybrids, the combination of two divergent genomes generally has a greater impact on the fish transcriptome than the presence of an additional set of chromosomes [23,24,25,26]. In the case of distant hybridization, one of the most pronounced outcomes observed in fish is the transcriptomic shock, characterized by a sudden and substantial shift in the genome-wide expression patterns [26,27,28,29]. Accumulating evidence indicates that the observed transcriptomic dysregulation in distant salmonid fish hybrids is mainly associated with significant under-expression of genes involved in energy metabolism, proteostasis, and stress response [30,31,32,33]. On the other hand, expression of genes involved in the same processes was found to be upregulated in autotriploids [34,35,36,37,38,39]. In turn, the downregulated genes in autotriploid fish are linked with cellular division processes, reflecting cell cycle disruptions caused by the presence of an odd chromosome number in their nuclei [24,35,36,37].

The regulation of gene expression in allotriploids, along with their physiology, is very complex and warrants study not only in the context of allopolyploid evolution but also for their incorporation into modern aquaculture. Examination of the gene expression in triploid hybrids has been studied to date mainly among closely related cyprinids [23,24,25,26] and *Epinephelus* fish species [28,29]. To extend our knowledge of how distant hybridization and triploidization reshape transcription of genes involved in core metabolic processes in salmonid hybrids, we decided to study, in this regard, diploid and triploid rainbow trout (*Oncorhynchus mykiss*), brook trout (*Salvelinus fontinalis*), and their crosses. Diploid hybrids of these species die before yolk-sac resorption due to the genomic incompatibilities; however, their viability can be rescued by the artificial triploidization [22]. Beyond the strictly scientific dimension, the study may also have practical value, as triploid hybrids of rainbow trout and brook trout, due to their increased resistance to VHS and IHN viruses, are considered promising candidates for commercial aquaculture [40,41,42]. We hypothesized that triploidization would attenuate the transcriptomic shock in rainbow trout × brook trout hybrids, thereby stabilizing the expression of genes involved in core metabolic and stress response processes associated with their physiological performance. Therefore, we selected a panel of ten representative genes based on three main criteria: (1) membership in functional clusters strongly associated with processes critical for hybrid viability, such as energy metabolism, proteostasis, ribosome biogenesis and stress response (2) evidence from previous studies indicating fold changes exceeding two between inviable diploid and viable triploid crosses and (3) repeated identification across multiple independent RNA-seq datasets on distant salmonid hybrids [33,34,35,36]. Transcriptional patterns of these genes were analyzed alongside the growth performance of triploid rainbow trout × brook trout hybrids reared for two years to provide preliminary data on the fitness of the examined hybrids under aquaculture conditions, thereby laying the groundwork for future physiological studies.

## 2. Materials and Methods

### 2.1. Fish Origin and Maintenance

The purebred diploid (2n RT) and triploid rainbow trout (3n RT), diploid brook trout (2n BT) and triploid crosses between rainbow and brook trout (3n RT × BT) examined in this study were produced through the controlled reproduction of fish originating from domesticated broodstocks maintained at the Department of Salmonid Research (DSR) of the Inland Fisheries Institute (IFI) in Olsztyn, Rutki (Poland) (54°19′51.1″ N 18°20′15.1″ E). Diploid RT × BT hybrids were not included in the analysis because they are completely inviable, exhibiting 100% mortality until yolk-sac resorption. All spawners were reared in ponds with a capacity of 10–30 m^3^ and a water flow of 5 dm^3^/s. The mean water temperature was 10 °C, and the oxygenation level exceeded 80% of the maximal oxygen concentration at a given temperature. The fish were fed with commercial broodstock feed (Aller Bronze 3 mm and Aller Silver 3 mm, Aller Aqua, Golub-Dobrzyń, Poland) three or four times daily in amounts ranging from 0.5 to 1.5% fish biomass, depending on water temperature [43]. Fish feeding was stopped two weeks before the spawning period. All gamete donors used were 3–4 years old and had an average body weight of 1.5–2.5 kg.

For each produced cross type, gametes were obtained from different sets of five females (egg donors) and three males (milt donors). Gamete collection was conducted during the natural spawning season in October. Prior to gamete stripping, selected spawners were anesthetized with MS-222 (50 mg/dm^3^, Sigma-Aldrich, Barcelona, Spain). A pooled-mating design approach was employed to minimize potential family effects and to generate representative offspring for each experimental group (2n RT, 3n RT, 2n BT, 3n RT × BT). For this purpose, eggs stripped from every five females were pooled into a single composite batch per group and immediately fertilized with collected milt from three males at a ratio of approximately 150,000 spermatozoa per egg. To produce diploid and triploid rainbow trout (2n RT and 3n RT) stocks, milt from hormonally induced neomales was used to generate all-female stocks. In turn, to produce diploid brook trout (2n BT) and triploid hybrids (3n RT × BT), normal milt from sexually mature brook trout males was used, resulting in mixed-sex stocks. Before fertilization, spermatozoa quality from each male was checked by direct observation of milt activated with sperm activating medium (SAM) (1 mM CaCl_2_, 20 mM Tris, 30 mM glycine, 125 mM NaCl, pH 9.0) [44] under an optical microscope (Nikon Eclipse E2000, Nikon Corporation, Tokyo, Japan) at a total magnification of 100×. After fertilization, selected batches of rainbow trout eggs (i.e., 3n RT and 3n RT × BT) were subjected to triploidization using a high hydrostatic pressure (HHP) treatment. The applied triploidization protocol involved a five-minute exposure of fertilized rainbow trout eggs to an HHP shock of 9500 psi applied 350 degree minutes (CTMs) after activation [45]. The HHP shock was generated using the TRC-APV electric/hydraulic device (TRC Hydraulics Inc., Dieppe, NB, Canada).

All obtained batches of eggs were incubated for about six weeks at a temperature of 7.0 ± 0.5 °C, with oxygen levels maintained at 10.8 ± 0.5 mg/L and the pH at 7.5 ± 0.1, in separate vertical incubators with egg trays. Hatched fish were reared under identical conditions in separate tanks and ponds supplied with water from the Radunia River (mean temperature of 10 °C and oxygenation levels above 80%) for about two years and six months (82 weeks). After yolk-sac resorption, fry were stocked into 0.3 m^3^ plastic tanks with a water flow rate of 1 liter per second (L/s). When the fish attained a mean body weight of 5 and 100 g/indiv., they were transferred to 1 m^3^ and 3 m^3^ tanks, respectively, with a flow rate of 4 L/s. After reaching 300 g/indiv., approximately 200 fish were reared in 10 m^3^ concrete rectangular ponds with a flow rate of 6 L/s, resulting in a stocking density of ~6 kg/m^3^. The fish were fed commercial feed formulated for salmonids (Aller Aqua, Golub-Dobrzyń, Poland) three to four times daily in accordance with rainbow trout feeding recommendations at specific temperatures [46]. During the rearing phase, fish were fed manually until they attained a mean body weight of 10 g, after which automatic timer feeders (FIAP 1520, Fiap Company, Ursensollen, Germany) were used. Feeding ceased during periods when the water temperature exceeded 20 °C.

After about two years and six months post-hatch, 40 randomly selected females from each fish stock were weighed using a Radwag C315.60.C2.M warehouse scale (±1 g) and measured by a scale (±1 mm) for total body weight (BW) and length (BL). In the case of diploid brook trout (2n BT) and triploid hybrid (3n RT × BT) groups, the stocks were mixed-sex, and female individuals were identified based on morphological sexual dimorphism and subsequently confirmed by molecular verification. To verify the genetic sex, hybrid status, and ploidy level of the examined fish, pelvic and adipose fin clips were collected from all individuals included in the analysis per experimental group. Fin clips were preserved in 96% ethanol and stored at 4 °C until genetic material extraction, while adipose fin clips were immediately used for fish ploidy analysis. Subsequently, eight randomly selected individuals (biological replicates) per group, with confirmed ploidy level and molecularly verified female sex, were selected for transcriptomic analyses. This sample size is consistent with previous qPCR-based gene expression studies in salmonids and represents a compromise between ethical minimization of animal use and the logistical constraints of processing multiple experimental groups. All fish selected for transcriptomic analysis were molecularly verified as females to ensure the reliability of the study material and eliminate potential sex-biased variability in the expression of analyzed genes. Before sampling, the fish were humanely sacrificed by cutting the spinal cord. Liver and muscle tissues (approximately 0.5 cm^3^) were immediately collected, submerged in RNAlater™ solution (Thermo Fisher Scientific, Waltham, MA, USA), incubated at 4 °C for 24 h, and finally stored at −24 °C until further analyses. In total, 16 tissue samples per group were collected, including liver and muscle tissue from each of the eight fish.

### 2.2. Ploidy, Genetic Sex, and Hybridization Verification

The ploidy level of the examined fish was confirmed with a CyFlow^®^ Ploidy Analyzer (Sysmex Partec GmbH, Görlitz, Germany) equipped with CyView^TM^ software (ver.1.8.0.82). For this purpose, collected adipose fins were processed with a CyStain^TM^ UV Precise T kit (Sysmex Partec GmbH, Germany). Before analysis, about 10 mg of fin material was minced, incubated for 5 min in Extraction Buffer, and then stained with DAPI Staining Buffer. The applied flow cytometry protocol is routinely used to verify the effectiveness of triploidy induction in fish, enabling confirmation of the expected 2:1 genomic DNA content ratio in triploid hybrids relative to diploid rainbow and brook trout controls.

To verify the genetic sex and hybrid status of the examined fish, PCR-based DNA genotyping was carried out. Genomic DNA was isolated from collected fin clips using the standard Chelex-100 method [47]. The quality and quantity of the isolated genomic DNA were checked using a NanoDrop™ One spectrophotometer (Thermo Fisher Scientific, Waltham, MA, USA). The genetic sex of the sampled fish was verified by PCR duplex amplification of the Y-chromosome-linked DNA marker (*sdY*) (sdY E2S1: CCCAGCACTGTTTTCTTGTCTCA, sdY E2AS2: CTGTTGAAGAGCATCACAGGGTC, and sdY E2AS4: CTTAAAACCACTCCACCCTCCAT) and 18S rDNA (18S S: GTYCGAAGACGATCAGATACCGT, 18S AS: CCGCATAACTAGTTAGCATGCCG) as a positive control [48]. In turn, the hybrid status of the progeny obtained from crossing rainbow trout and brook trout was verified by applying the *OMM-1279* microsatellite DNA marker (F: GCGTGCCTGTTTGTTCATAGATG, R: CTGCCTGCAGAGAGCTGAGTAGTT) [49]. The PCR amplifications were carried out using 10 ng of isolated DNA template in a reaction mixture with a total volume of 12.5 μL, composed of 1 × GoTaq^®^ Hot Start Green Master Mix (Promega, Madison, WI, USA), as well as 0.4 μM of each *SdY* primer and 0.1 μM of each 18S rDNA primer (genetic sex screening) or 0.3 μM of each *OMM-1279* primer (hybrid status verification). PCR amplification was performed with a Mastercycler^®^ X50s thermocycler (Eppendorf, Hamburg, Germany) under the following conditions: an initial denaturation at 96 °C for 4 min, followed by 35 cycles of 94 °C for 30 s, annealing at 60 °C for 45 s, elongation at 72 °C for 45 s, and a final elongation step at 72 °C for 10 min. The resulting PCR products were separated on a 2.0% agarose gel (Sigma-Aldrich, St. Louis, MO, USA) stained with ethidium bromide (0.05 mg/mL) and then visualized using a UV transilluminator (Vilber Lourmat ECX-20.M, Eberhardzell, Germany).

### 2.3. RNA Extraction, cDNA Synthesis, and Real-Time PCR Analysis

Total RNA from tissues preserved in RNAlater solution was extracted using the Bead-Beat Total RNA Mini Kit (A&A Biotechnology, Gdańsk, Poland), following the manufacturer’s instructions. Residual DNA in the extracted RNA samples was removed using the Clean-Up Concentrator Kit (A&A Biotechnology, Gdańsk, Poland). RNA concentration and purity were measured with a NanoDrop One spectrophotometer (Thermo Fisher Scientific, Waltham, MA, USA), and RNA integrity was assessed by 1% agarose gel electrophoresis. The obtained RNA samples were immediately processed further.

Purified total RNA samples of satisfactory quality were used to synthesize cDNA using the RevertAid First Strand cDNA Synthesis Kit (Thermo Fisher Scientific, Waltham, MA, USA). The reaction mixtures were prepared in a total volume of 20 μL, composed of 1× Reaction Buffer, 5 μM Random Hexamer primer, 1 μM dNTP Mix, 20U RiboLock RNase Inhibitor, 20U RevertAid M-MuLV RT reverse transcriptase, and 1 μg RNA sample. The reverse transcription reactions were carried out on a Mastercycler^®^ X50a (Eppendorf, Germany). The samples were incubated for 5 min at 25 °C, followed by 60 min at 42 °C, and the reaction was terminated by heating at 70 °C for 5 min. The obtained cDNA samples were diluted 1:10 with DEPC-treated water and stored at −20 °C for further processing.

Transcriptomic analyses included ten target genes involved in five critical biological processes important for the viability and fitness of salmonid fish hybrids, namely: (1) cellular respiration and energy production (*ATP synthase peripheral stalk-membrane subunit b* and *ADP/ATP translocase 2*), (2) proteasome-mediated protein turnover (*Derlin1* and *26S proteasome regulatory subunit 7*), (3) ribosome biogenesis (*60S Ribosomal Protein L24* and *40S ribosomal protein S24*), (4) mitochondrial functions (*mitochondrial ribosomal protein L28* and *mitochondrial calcium uptake protein 2*) and (5) protein chaperoning (*heat shock protein 90 β family member 1* and *protein disulfide-isomerase A4*). The primer sequences for the analyzed genes were designed based on genetic information deposited for rainbow trout and brook trout in the GenBank (release 101) and Ensembl (release 109) databases or derived from the available literature (Table 1). All available isoforms and splice variants of the analyzed genes were taken into consideration before primer construction, and the primers were designed to allow simultaneous transcription analysis in both species. To eliminate inter-specific sequence variation as a source of differential gene expression estimates, conserved gene regions between rainbow trout and brook trout were identified and considered during the primer design. The primers were designed with default parameters, including placement across exon–exon junctions and a qPCR product length of approximately 100–200 bp, and checked for complementarity with the genomes of both species using the Primer-Blast online tool (NCBI). All primers were also amplified in both rainbow trout and brook trout samples, sequenced using a 3730xl DNA Analyzer (Applied Biosystems, Carlsbad, CA, USA), and verified against the GenBank nucleotide database using the online NCBI BLAST tool (https://blast.ncbi.nlm.nih.gov/Blast.cgi) to verify that all primers amplified the correct gene transcript targets. This approach confirmed the absence of differential amplification between the parental species, ensuring that all primers accurately amplify the corresponding transcripts in both rainbow trout and brook trout.

Real-Time PCR analysis was carried out using designed primers for the target genes, choosing β-actin (*Actb*) (F: GCCGGCCGCGACCTCACAGACTAC, R: CGGCCGTGGTGGTGAAGCTGTAGC) and elongation factor 1-alpha (*Elf1a*) (F: TTAAGCAACCATGGGAAAGG, R: TACCTGCCGGTCTCAAACTT) as the housekeeping genes due to their proven stable expression levels across different tissues [41,53,54,55]. Moreover, numerous studies indicate that the mentioned reference genes show a 1:1 dosage effect between triploid and diploid fish and maintain stable expression levels in hybrids between rainbow trout and other salmonids, making them suitable as internal controls for relative mRNA transcription analyses in the fish examined in this study [11,56,57,58,59,60]. The stability of the used reference genes was also confirmed in the examined fish groups via pairwise relative gene expression analyses, yielding M stability values of 0.844 for both *Actb* and *Elf1a*, well below the commonly accepted threshold of *M* < 1.5. The qPCR was performed on a Cielo 6 Real-Time PCR System (Azure Biosystems, USA) using the PowerTrack SYBR Green Master Mix (Applied Biosystems, CA, USA). Reaction efficiencies were estimated from the slopes of the standard curves made of 10-fold serial cDNA dilutions starting from about 20 ng/μL and were verified individually for each target and reference gene, with all assays displaying efficiencies between 90 and 110%. The qPCR reaction mixtures were prepared in a total volume of 10 μL, consisting of 1× PowerTrack SYBR Green Master Mix, 0.5–0.8 μM (target genes) and 0.15–0.5 μM (housekeeping genes) of each primer, as well as 5 ng cDNA. The Real-Time PCRs were run in triplicate with the following thermal cycling conditions: an initial polymerase activation step at 95 °C for 5 min, followed by 35 cycles of 95 °C for 30 s (denaturation), 20 s at 60 °C (primer annealing), and 72 °C for 15 s (elongation). During each run, negative controls using pure water and non-transcribed RNA were used to exclude reagent and sample contamination. The analysis of the melting curve (60–95 °C) at the end of each run concluded the protocol. Fluorescence data were collected after the elongation step and in 0.1 °C steps on the melting curve. Relative expression levels of target genes were calculated using the ΔΔCt method described by Livak and Schmittgen [61]. First, Ct values from technical triplicates were averaged for each sample. Then, averaged Ct values of the reference genes for each sample were normalized to the geometric mean using the geNorm software (ver. 3.4) [62]. ΔCt was calculated as Ct_target − Ct_ref, and ΔΔCt as ΔCt_sample − ΔCt_calibrator, where the calibrator was the mean ΔCt of the diploid rainbow trout (2n RT) samples for the same tissue. Fold change in expression is reported as 2^−ΔΔCt^.

### 2.4. Statistical Analysis

Statistical analyses of the fish body weight and length, as well as fold change expression values for target genes relative to the normalized expression levels of reference genes (*ß-actin* and *Elf1a*) were carried out using Statistica software (ver.10.0, StatSoft Inc., Tulsa, OK, USA). Prior to analysis, the normality of data distribution and homogeneity of variances were assessed using the Shapiro–Wilk test and Levene’s test, respectively. All datasets were confirmed to follow normal distribution and homogeneity of variances, supporting the assumption that the chosen sample size was representative for each experimental group. Moreover, a post hoc power analysis based on the recorded variability in ΔCq values within each fish group (SD range 0.29–1.88 across genes and tissues) indicated that the sampling design was sufficiently powered to detect large effect sizes (f ≈ 0.40, power ≈ 87%), allowing the identification of strong, biologically meaningful gene expression changes.

Differences in gene expression were analyzed using a two-way ANOVA with fish group (2n RT, 2n BT, 3n RT, and 3n RT × BT) and tissue (liver and muscle) as fixed factors, including tests for the interaction effect (fish group × tissue). Post hoc Tukey’s HSD tests, which account for multiple comparisons, were applied to determine significant differences between tissues within each fish group and between fish groups within each tissue (*p* < 0.05).

Mid-parent heterosis (MPH, %) for body weight and body length was calculated to assess growth performance in the examined rainbow trout × brook trout hybrids using the formula:

$$MPH\;(\%)=\frac{Hybrid\;mean-Midparent\;mean}{Midparent\;mean}\times 100$$

where the mid-parent mean represents the average of the two diploid parental species (2n RT and 2n BT). Moreover, mean values of Fulton’s condition factor (*K*) were calculated for each fish group, using the following formula:

$$K=\frac{body\;weight\;(g)}{{body\;length\;(cm)}^{3}}\times 100$$

## 3. Results

### 3.1. Growth Performance of Examined Fish

**Figure 1:**
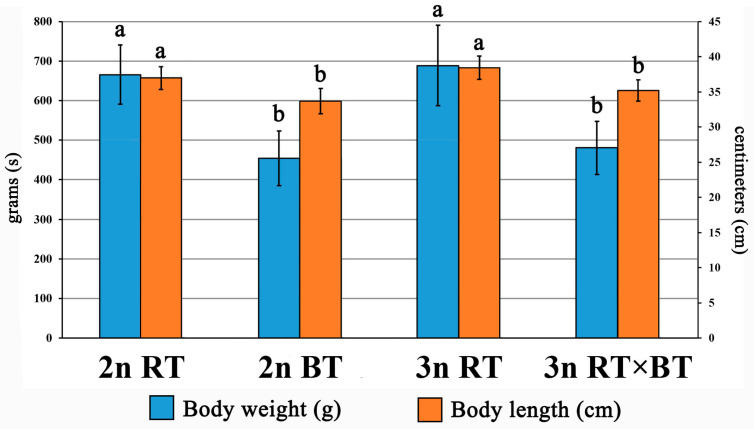
Average body weight (BW) and body length (BL) recorded for diploid (2n RT) and triploid rainbow trout (3n RT), diploid brook trout (2n BT), as well as triploid hybrids between rainbow trout × brook trout (3n RT × BT) examined in the present study after about two and a half years of rearing under identical aquaculture conditions. Different letters indicate statistically significant (*p* < 0.05) differences in the recorded values of the analyzed morphological parameters between the examined fish groups. Data is shown as the means with standard deviations (±SD).

After the experimental rearing period, the highest average body weight and length were observed in purebred rainbow trout, both triploid (3n RT, BW = 689 ± 77 g, BL = 38.3 ± 1.7 cm, *K* = 1.21) and diploid (2n RT, BW = 666 ± 75 g, BL = 37 ± 1.6 cm, *K* = 1.32). In contrast, the lowest growth parameters were recorded in purebred diploid brook trout (2n BT, BW = 454 ± 50 g, BL = 33.7 ± 1.4 cm, *K* = 1.19) and triploid hybrids (3n RT × BT, BW = 481 ± 68 g, BL = 35.2 ± 1.5 cm, *K* = 1.10). Statistically significant differences (*p* < 0.05) in both traits were found between the group of purebred rainbow trout (2n RT, 3n RT) and the group comprising diploid purebred brook trout together with triploid hybrids (2n BT, 3n RT × BT). No significant differences (*p* > 0.05) were detected between diploid (2n RT) and triploid (3n RT) purebred rainbow trout, nor between purebred diploid brook trout (2n BT) and triploid hybrids (3n RT × BT) (Figure 1). The calculated mid-parent heterosis (MPH) index for the examined triploid hybrids revealed negative values (MPH = −14.3%) for body weight and (MPH = −0.4%) for body length, indicating that the hybrids grew slightly less than the mid-parent mean of their purebred parental species.

### 3.2. Ploidy and Hybridization Confirmation

**Figure 2:**
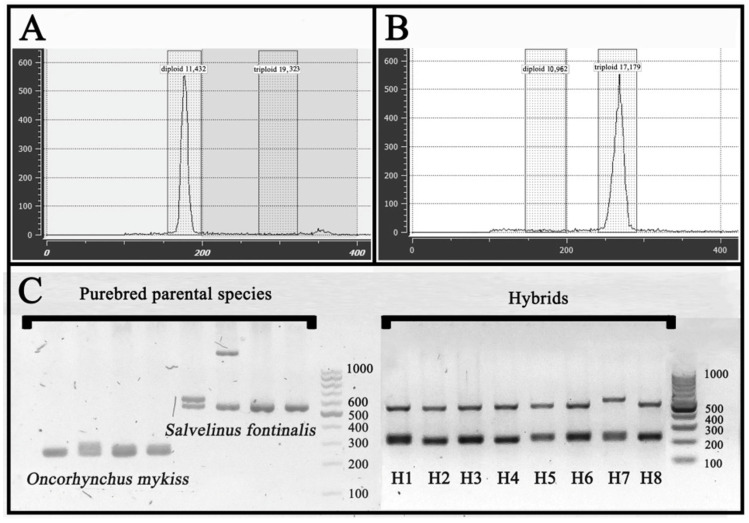
Results of ploidy verification and hybrid status confirmation of the fish used in the present study. Flow cytometry profiles of cellular DNA content in (**A**) diploid (2n) and (**B**) triploid (3n) individuals sampled. (**C**) genotyping results of triploid rainbow trout × brook trout (*Salvelinus fontinalis*) hybrids (lanes H1–H8) and purebred parental species (reference) by PCR amplification of the *OMM-1279* microsatellite DNA marker. DNA size marker: 100 bp DNA ladder (A&A Biotechnology s.c., Poland).

Flow cytometry analysis confirmed the ploidy level of diploids (2n RT and 2n BT) and triploids (3n RT and 3n RT × BT) as expected (Figure 2A,B). The amplification of *OMM-1279* microsatellite DNA marker yielded products of length 200–300 base pairs (bp) for rainbow trout and 550–1500 bp for brook trout (Figure 2C). Molecular screening conducted on all sampled fish revealed genotypes originating from both parental species, confirming their hybrid status. Moreover, the PCR-based DNA genotyping confirmed that all sampled fish were genetically female (Supplementary Figure S1).

### 3.3. Gene Expression Analysis

**Figure 3:**
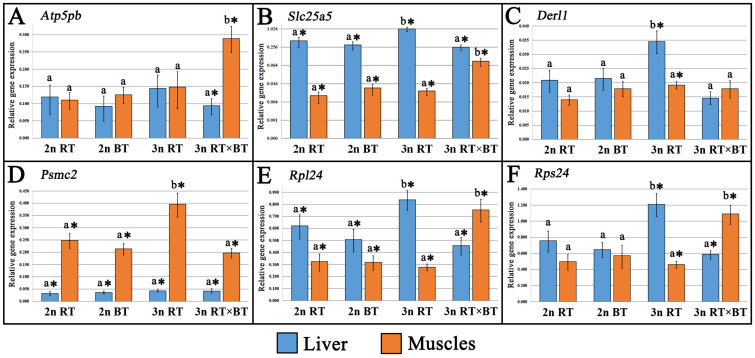
Relative expression levels of (**A**) ATP synthase peripheral stalk-membrane subunit b (*Atp5pb*), (**B**) ADP/ATP translocase 2 (*Slc25a5*), (**C**) Derlin1 (*Derl1*), (**D**) 26S proteasome regulatory subunit 7 (*Psmc2*), (**E**) 60S Ribosomal Protein L24, (*Rpl24*) and (**F**) 40S ribosomal protein S24 (*Rps24*) in the liver and muscle tissues sampled from diploid (2n RT) and triploid rainbow trout (3n RT), diploid brook trout (2n BT), as well as triploid hybrids between rainbow trout × brook trout (3n RT × BT) examined in the present study. Distinctive letters indicate statistically significant (*p* < 0.05) differences in the recorded gene expression levels between the examined fish groups within each tissue type. Asterisks indicate statistically significant (*p* < 0.05) differences in gene expression levels between tissues within each fish group. Expression values were calculated relative to the diploid rainbow trout (2n RT) group, which served as the calibrator in the ΔΔCt method. Data are presented as mean relative expression values (2^−ΔΔCt^) normalized to the reference genes (*β-actin* and *elongation factor 1-alpha*), with variation shown as ±SEM calculated from biological replicates. Relative expression values on some figures are presented on a log_10_ scale to improve visualization of differences across genes with varying expression levels.

**Figure 4:**
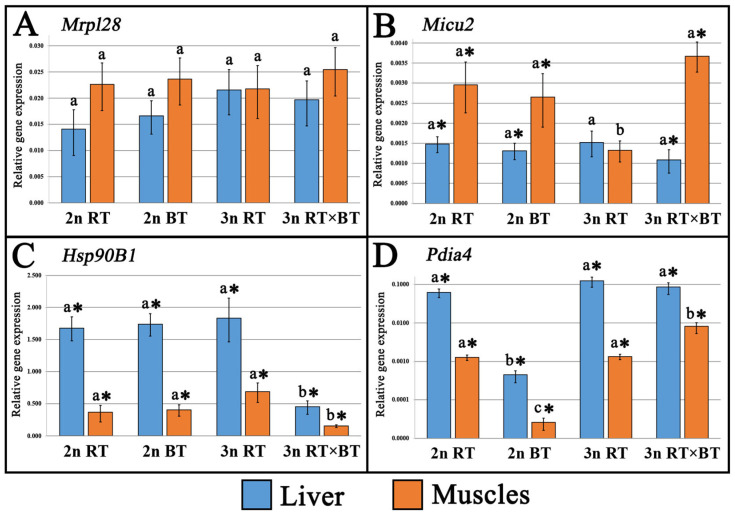
Relative expression levels of (**A**) Mitochondrial ribosomal protein L28 (*Mrpl28*), (**B**) Mitochondrial calcium uptake protein 2 (*Micu2*), (**C**) Heat shock protein 90 β family member 1 (*Hsp90B1*), (**D**) Protein disulfide-isomerase A4 (*Pdia4*) in the liver and muscle tissues sampled from diploid (2n RT) and triploid rainbow trout (3n RT), diploid brook trout (2n BT), as well as triploid hybrids between rainbow trout × brook trout (3n RT × BT) examined in the present study. Distinctive letters indicate statistically significant (*p* < 0.05) differences in the recorded gene expression levels between the examined fish groups within each tissue type. Asterisks indicate statistically significant (*p* < 0.05) differences in gene expression levels between tissues within each fish group. Expression values were calculated relative to the diploid rainbow trout (2n RT) group, which served as the calibrator in the ΔΔCt method. Data are shown as mean relative expression values (2^−ΔΔCt^) normalized to the reference genes (*β-actin* and *elongation factor 1-alpha*), with variation presented as ±SEM calculated from biological replicates. Relative expression values on some figures are presented on a log_10_ scale to enhance visualization of differences across genes with varying expression levels.

The mRNA expression for each investigated gene was detected in every tissue sampled from all examined fish (Figure 3 and Figure 4). Two-way ANOVA revealed significant (*p* < 0.05) fish group × tissue interactions for *Slc25a5*, *Derl1*, *Micu2*, *Pdia4*, *Psmc2*, *Rpl24* and *Rps24*, indicating that the effect of ploidy and hybridization on transcription levels depended on tissue type, whereas no significant (*p* > 0.05) interaction was detected for *Atp5pb*, *Hsp90B1* and *Mrpl28*, suggesting consistent group-related patterns across tissues.

In the liver tissue, significantly (*p* < 0.05) higher mRNA transcription levels of *Slc25a5* (η^2^ = 0.46), *Derl1* (η^2^ = 0.51), *Rps24* (η^2^ = 0.50) and *Rpl24* (η^2^ = 0.33) were recorded in triploid purebred rainbow trout when compared to diploid rainbow and brook trout, as well as triploid hybrids (Figure 3B,C,E,F). Insignificant (*p* > 0.05) differences in gene expression levels among all studied fish groups were observed for *Atp5pb*, *Psmc2*, *Mrpl28*, and *Micu2* genes in the liver (Figure 3A,D and Figure 4A,B). For the *Pdia4* gene, significant (*p* < 0.05, η^2^ = 0.34) differences in transcription levels were observed only in diploid brook trout, where expression was approximately three-fold lower than in all other fish groups (Figure 4D).

In muscle tissue, significant (*p* < 0.05) upregulation of mRNA transcription level was detected in triploid hybrids for most of the examined genes, namely: *Atp5pb* (η^2^ = 0.25), *Slc25a5* (η^2^ = 0.65), *Rpl24* (η^2^ = 0.51), *Rps24* (η^2^ = 0.49) and *Pdia4* (η^2^ = 0.65), while slightly higher (*p* > 0.05) expression levels were observed in triploid rainbow trout compared with diploid rainbow trout and brook trout (Figure 3A,B,E,F and Figure 4D). In the case of the *Psmc2* gene, significantly higher (*p* < 0.05, η^2^ = 0.59) expression levels were recorded in triploid rainbow trout, when compared to all other fish groups (Figure 3D). Among all analyzed genes, significant (*p* < 0.05, η^2^ = 0.46) downregulation of expression was detected for the *Micu2* gene in triploid rainbow trout (Figure 4B). No significant (*p* > 0.05) differences in gene expression levels between the studied fish groups were observed for *Derl1* and *Mrpl28* genes in the muscle tissue (Figure 3C and Figure 4A).

Three- to four-fold lower (*p* < 0.05, η^2^ = 0.49–0.65) expression levels of the *Hsp90B1* gene were detected in both tissues of triploid hybrids compared with diploid and triploid rainbow trout, as well as diploid brook trout. The reduced expression was consistent across all analyzed individuals within the triploid hybrid group and was not driven by outlier values. Compared to diploid rainbow trout and brook trout specimens, slightly upregulated *Hsp90B1* transcription levels were recorded in triploid rainbow trout; however, the observed differences were not significant (*p* > 0.05) in both examined tissues (Figure 4C).

## 4. Discussion

Interspecific hybridization and triploidization are important reproductive biotechnologies in aquaculture [63,64,65], leading to rapid genotypic modifications, as well as pronounced physiological and phenotypic changes in the resulting progeny [66,67,68]. In the present study, the effects of hybridization and triploidization on the expression patterns of ten selected genes involved in pathways related to cellular respiration and energy production, proteasome-mediated protein turnover, ribosome biogenesis, mitochondrial functioning, as well as protein chaperoning were examined in the liver and muscle tissues of triploid (3n) crosses between rainbow trout and brook trout that are considered promising for commercial aquaculture production. Flow cytometry analysis and DNA genotyping confirmed that the examined fish were triploid hybrids between the aforementioned species (Figure 2A,B).

One of the key outcomes of additional chromosome sets in polyploids is the genome dosage effect, which results in gene expression levels proportional to gene copy number [69,70,71]. Unlike mammals, fish adapt well to the genome alterations caused by ploidy changes because of their ability for functional rediploidization [24,72,73,74]. The observed stabilization of genome expression in polyploid fishes toward a diploid state is achieved through gene dosage compensation [73,74,75,76,77,78,79]. Available studies suggest that dosage compensation in triploids involves proportional silencing of each gene copy, with all three remaining transcriptionally active in an additive pattern, typically resulting in genome-wide homeolog expression dominance (HED) and bias (HEB) toward the maternal genome [68,80,81,82,83]. In this study, most analyzed genes, i.e., *Slc25a5*, *Derl1*, *Rpl24*, *Rps24* (in muscles), *Psmc2*, *Micu2* (in the liver), *Atp5bp*, *Mrpl28*, *Hsp90B1,* and *Pdia4* (in both tissues), exhibited dosage compensation in purebred triploid rainbow trout, with expression levels comparable to their diploid counterparts (Figure 3 and Figure 4). Among the mentioned genes, the *Atp5pb*, *Micu2*, *Pdia4* (in the liver tissue), *Derl1* (in the muscles), as well as *Mrpl28* and *Psmc2* (in both liver and muscles) also displayed unchanged expression levels in the triploid rainbow × brook trout hybrids compared with diploid rainbow trout and brook trout, suggesting their potential role in maintaining key physiological functions.

In polyploids, genome dosage compensation mechanisms do not always precisely match expression levels to those observed in diploids, as some genes exhibit significant upregulation or downregulation, which is referred to as positive and negative dosage compensation [73,84]. Intriguingly, all genes that displayed positive (*Slc25a5*, *Rpl24*, *Rps24*, *Derl1,* and *Psmc2*) or negative (*Micu2*) dosage compensation in the tissues of purebred triploid rainbow trout relative to their diploid counterparts also exhibited comparable expression levels between the examined triploid hybrids and the purebred diploid parental species (Figure 3B–F and Figure 4B). Given that diploid hybrids between rainbow and brook trout are inviable and die before yolk-sac resorption [22], the induction of triploidization in the examined hybrids may alleviate the severity of transcriptomic shock caused by merging genomes from different species. Indeed, an accumulating number of studies indicate that whole-genome duplications following hybridization can weaken the postzygotic reproductive constraints, enhancing viability and fitness of allopolyploid fishes [85,86,87,88,89]. Available research indicates that the observed reduction in transcriptomic dysregulation in allopolyploid fishes primarily stems from increased gene expression flexibility, driven by variable dosage compensation, which involves the silencing of *cis*-regulatory elements and the enhancement of *trans*-regulatory genomic mechanisms [24,25,26,73]. Since the examined triploid hybrids inherit two chromosome sets from rainbow trout (2n) and one from brook trout (1n), their increased viability may be associated with the unequal genomic contribution of parental species, as suggested by previous studies on autotriploid salmonid fishes [80,81]. Numerous studies have revealed that allotriploid fishes retain a larger portion of the maternal genome, including maternal-specific genes together with patterns of genome methylation and transposable-element density, which reduces incompatibilities between nuclear and mitochondrial genomes in hybrid offspring, thereby alleviating the severity of potential cytonuclear conflicts that could impair core cellular physiological processes [68,86,90,91,92,93].

The first generation of fish hybrids typically exhibits enhanced trait performance, a phenomenon known as heterosis or transgressive inheritance [1,2,88]. The observed trait superiority in distant fish crosses over purebred parental species is usually linked to enhanced growth parameters [56,94,95], improved innate immune responses [96,97], and increased resistance to unfavorable environmental conditions [98,99]. Available studies indicate that heterosis in allopolyploid fishes is mainly due to increased heterozygosity, gene redundancy, incomplete dosage compensation, and maternal or paternal dominance effects [80,96,100]. Multiple transcriptomic studies have linked the upregulation of genes associated with protein synthesis and energy metabolism to growth heterosis in allopolyploid fishes [23,73,101]. Significant upregulation of genes involved in protein synthesis (*Rpl24*, *Rps24,* and *Pdia4*) and energy-production (*Atp5pb*, *Slc25a5,* and *Micu2*) was detected in the muscles of the examined triploid hybrids between rainbow and brook trout (Figure 3A,B,E,F and Figure 4B,D). However, the recorded morphological data indicated that growth performance did not show clear evidence of heterosis in the examined triploid hybrids (Figure 1). Similar divergent patterns between transcriptional activity and growth performance in triploid salmonid fishes have been linked to their metabolic inefficiencies associated with reduced mitochondrial density, lower ATP yield, or impaired oxygen transport capacity, possibly limiting their aerobic performance despite transcriptional upregulation of metabolic genes [36,37,38,102]. However, without direct physiological measurements, it remains unknown whether the recorded upregulation of energy-related genes in the examined triploid hybrids reflects enhanced metabolic efficiency or a compensatory response aimed at maintaining energy homeostasis. It is also important to note that extensive research on allotriploid and autotriploid salmonid fishes has highlighted the critical role of the maternal genome’s origin in shaping complex traits among hybrid families [80,81,103,104]. Therefore, further research to elucidate how the maternal origin of the genome influences growth rate, resistance to environmental stress and pathogens, survivability, as well as deformity rates in the triploid hybrids of rainbow trout × brook trout examined here, offers a viable opportunity to gain valuable information for the future improvement of their aquaculture production.

Interspecific hybridization and polyploidization are known to impose significant stress on organisms by causing molecular, chromosomal, and cellular changes that disrupt key processes essential for genomic stability, protein synthesis, and metabolic regulation [19,29,105,106,107]. In the case of autotriploid fishes, their normal development may depend on the upregulation of stress-response genes, which likely act as a compensatory mechanism to mitigate physiological (e.g., oxidative stress) and environmental (e.g., hypoxia, hyperthermia) challenges arising from their limited aerobic energy budget and reduced capacity to store energy reserves [19,75,76]. The *Hsp90B1* and *Pdia4* genes analyzed in this study are central to cellular protein homeostasis, ensuring their proper folding, stabilization after synthesis, and repair during thermal and oxidative stress [108,109,110]. Consistent with previous research [9,34,36,37,38], a slight but statistically non-significant upregulation of both genes was also observed in purebred triploid (3n) rainbow trout compared with diploid individuals (Figure 3C,D). Conversely, a three- to four-fold downregulation of *Hsp90B1* expression was observed in the liver and muscle tissues of the examined triploid hybrids compared with purebred diploid and triploid rainbow trout (Figure 4C). These findings are consistent with other studies on salmonid hybrids that have reported marked downregulation of stress-response genes, particularly those associated with protein folding and structural maintenance [34,35,36,111,112]. It is hypothesized that this downregulation may result from improper gene expression regulation or negative epistatic effects due to maladaptive genome incompatibilities between parental species [12,13,14]. Autotriploid fishes have also been commonly reported to exhibit impaired tolerance to high temperatures [112,113,114,115], which is known to be associated with their disrupted cellular homeostasis [19,77]. Taken together, the observed downregulation of the *Hsp90B1* gene in the liver and muscles of the triploid hybrids may indicate altered regulation of stress-response pathways; however, without direct thermal challenge experiments, no definitive conclusions regarding heat tolerance can be drawn, and further studies are required. Finally, although no tank effect was observed in our statistical model, the use of a single tank per group represents a potential limitation of the experimental design and should be considered when interpreting the results.

## 5. Conclusions

Most of the analyzed genes displayed comparable expression levels across all fish groups, showing dosage compensation in purebred triploid rainbow trout, as well as in triploid rainbow trout × brook trout hybrids. Moreover, stabilized expression of genes that were positively or negatively compensated in purebred triploid trout was also observed in the examined triploid hybrids. Although this pattern may indicate that the combination of hybridization and triploidization alleviates transcriptomic shock in the examined fish cross, further analyses of subgenome-specific expression, maternal transcriptional dominance, and epigenetic regulatory mechanisms are required to draw robust conclusions regarding genomic incompatibility and its molecular basis in the examined hybrids. Despite the upregulation of genes related to protein synthesis and energy metabolism in the muscles of triploid hybrids, no growth advantage over purebred rainbow trout or brook trout was recorded. Genes encoding heat shock proteins were three- to four-fold downregulated in both liver and muscle tissues of triploid hybrids. While this may suggest altered stress-response regulation, direct measurements of thermal tolerance or stress resistance were not performed, and the functional consequences of this pattern remain to be verified. Overall, these results provide preliminary insight into how hybridization and triploidization affect key physiological pathways in triploid crosses between rainbow and brook trout, laying the groundwork for future studies that may provide valuable information for improving their aquaculture production.

## Figures and Tables

**Table 1:** Forward (F) and reverse (R) primer sequences used for the real-time PCR analysis of ten selected genes in the present study.

Gene	Primer Sequences	Amplicon Size (bp)	Biological Function	References
*ATP synthase peripheral stalk-membrane subunit b* (*Atp5pb*)	F: AAGAAGGAGCAGTGGAGGGC R: CACCATGTGGACCCTCTCCC	108	ATP production, Oxidative phosphorylation	ENSOMYG00000009141, designed in this study
*ADP/ATP translocase 2* (*Slc25a5*)	F: GGATTCTCCGTGTCGGTCCA R: GGACGGTATCGAAGGGGTAGG	178	ADP/ATP transport, Energy homeostasis	ENSOMYG00000025595, designed in this study
*Derlin1* (*Derl1*)	F: AACTGATTGGGAACCTGGTGGG R: GTGGCGCTCCAAACCCAGAC	153	ER-associated protein degradation	ENSOMYG00000057377, designed in this study
*26S proteasome regulatory subunit 7* (*Psmc2*)	F: ATCAGGGTCATCGGCTCAGA R: GCCCCTCCAATAGCGTCAAT	137	26S proteasome regulatory subunit, Protein degradation	ENSOMYG00000008822, [50]
*60S Ribosomal Protein L24* (*Rpl24*)	F: CAAGAAGGGCCAGTCTGAAG R: CAGGCTTCTGGTTCCTCTTG	119	Ribosome assembly, Protein synthesis	ENSOMYG00000021073, [51]
*40S ribosomal protein S24* (*Rps24*)	F: AAACCGGCTGCTTCAGAGGAA R: GCCACCACCAAACTGTGTCC	160	Ribosome assembly, Protein synthesis	ENSOMYG00000047442, designed in this study
*Mitochondrial ribosomal protein L28* (*Mrpl28*)	F: CCAGGATGGCCTATGGGGAG R: GTCTAGTGCGCGAGCCGTTA	175	Mitochondrial ribosome, Mitochondrial protein synthesis	ENSOMYG00000021960, designed in this study
*Mitochondrial calcium uptake protein 2* (*Micu2*)	F: GACAGTGCCTAAGGAAGGTATCA R: ACCATTCCTGCCAAAGAAGAAGG	97	Mitochondrial calcium uptake, Metabolic regulation	ENSOMYG00000009310, designed in this study
*Heat shock protein 90 β family member 1* (*Hsp90B1*)	F: TTGCGTGGAACTAAGGTGA R: CCAATGAACTGAGAGTGCT	104	Molecular chaperone, Protein folding	ENSOMYG00000040017, [52]
*Protein disulfide-isomerase A4* (*Pdia4*)	F: ATGAGAAAGCTTCACACACGCT R: CACCAGTGGCAGGATGTGTTTC	92	ER chaperone, Disulfide bond formation	ENSOMYG00000007216, designed in this study

## Data Availability

The raw data supporting the conclusions of this article will be made available by the authors upon request.

## Supplementary Materials

The following supporting information can be downloaded at https://www.mdpi.com/article/10.3390/cimb48030320/s1.
