# Supplementary material for: Effects of Hybridization and Triploidization on Transcription of Core Metabolic and Stress Response Genes in Rainbow Trout (Oncorhynchus mykiss) × Brook Trout (Salvelinus fontinalis) Hybrids—Preliminary Results
Source: Curr Issues Mol Biol. 2026 Mar 17;48(3):320. doi: 10.3390/cimb48030320 (PMC13024855; doi:10.3390/cimb48030320)
Supplement: Supplementary file 1 [file cimb-48-00320-s001.zip › cimb-4162106-supplementary.pdf]

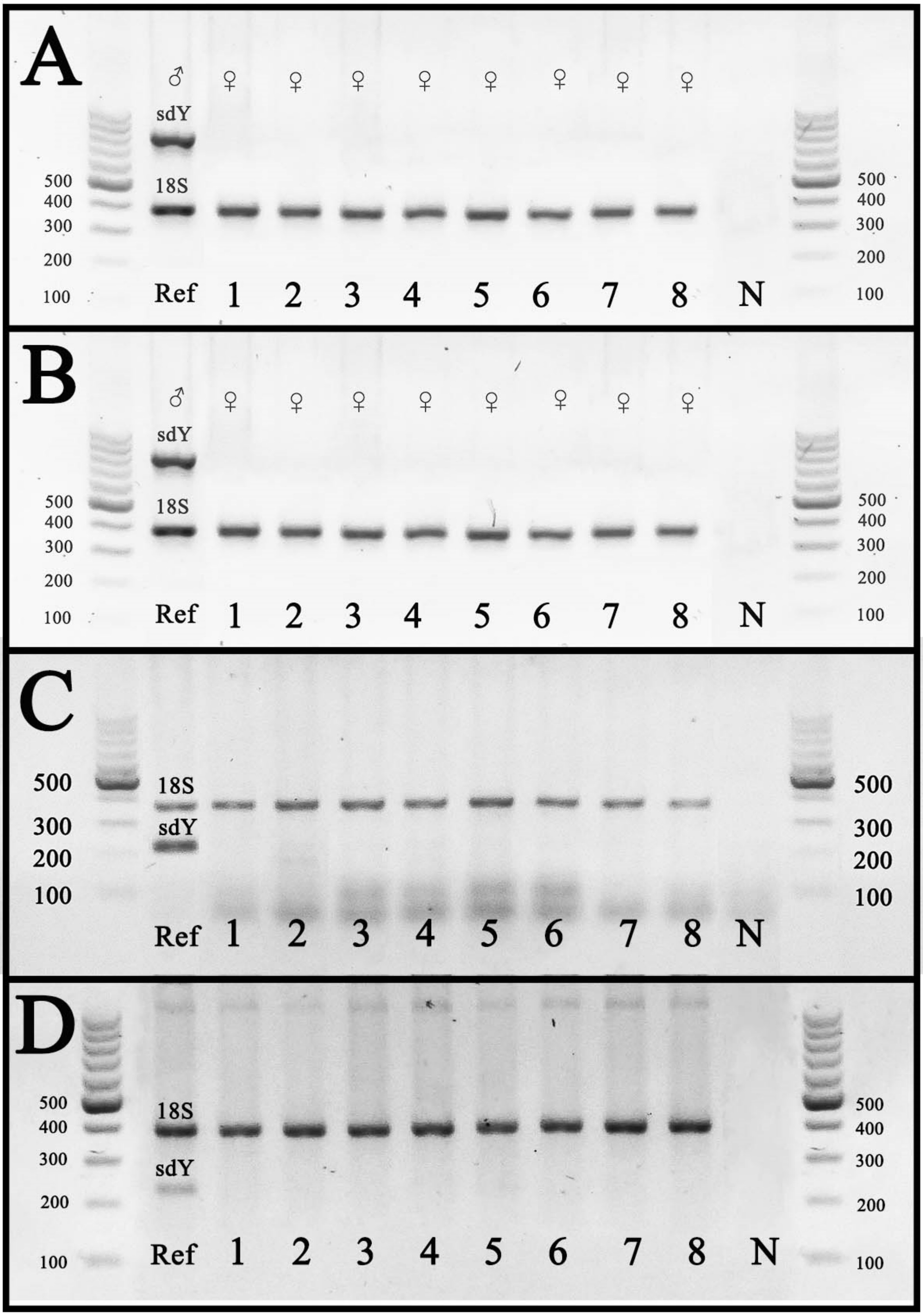

Figure S1. Sex genotyping of the diploid (A) and triploid (B) rainbow trout, triploid hybrids between rainbow trout × brook trout hybrids (C), as well as diploid brook trout (D) examined

in the current study by PCR multiplex amplification of Y chromosome-linked marker (sdY E2S1: CCCAGCACTGTTTTCTTGTCTCA, sdY E2AS2: CTGTTGAAGAG-CATCACAGGGTC and sdY E2AS4: CTTAAAACCACTCCACCCTCCAT) and 18S rDNA positive amplification control (18S S: GTYCGAAGACGATCAGATACCGT, 18S AS: CCGCATAACTAGTTAGCATGCCG). Ref: reference DNA template extracted from male individual of rainbow trout. N: negative control assay without DNA template. DNA weight marker: 100 bp DNA ladder (A&A Biotechnology s.c., Poland).
